# Precision-cut bovine udder slices (PCBUS) as an in-vitro-model of an early phase of infection of bovine mastitis

**DOI:** 10.1186/s12917-021-02817-w

**Published:** 2021-03-16

**Authors:** Viviane Filor, Monique Petry, Jessica Meißner, Manfred Kietzmann

**Affiliations:** 1 Department of Pharmacology, Toxicology and Pharmacy, University of Veterinary Medicine Hannover, Foundation, Bünteweg 17, 30559 Hannover, Germany; 2 Department of Veterinary Medicine, Institute of Pharmacology and Toxicology, Freie Universität Berlin, Koserstraße 20, 14195 Berlin, Germany

**Keywords:** Bovine mastitis, Cytokine profile, In-vitro-model, Precision-cut bovine udder slices

## Abstract

**Background:**

The aim of this study was to establish precision-cut bovine udder slices (PCBUS) as an in-vitro-model to investigate pathophysiological processes in the early phase of mastitis in order to have the possibility to investigate new therapeutic approaches for the treatment of such udder inflammation in later studies. Furthermore, this model should contribute to substitute in-vivo-experiments. Bovine mastitis is one of the most common and costly infectious diseases in the dairy industry, which is largely associated with the use of antimicrobial agents. Given this problem of antimicrobial resistance, it is essential to step up research into bacterial infectious diseases. Thus, the transfer of the in-vitro-model of precision-cut tissue slices to the bovine udder enables broad research into new therapeutic approaches in this area and can also be used to address issues in basic research or the characterisation of complex pathophysiological processes.

**Results:**

A stimulation with LPS, PGN or the combination of both substances (LPS:PGN) demonstrates the ability of the PCBUS to react with a significant secretion of IL-1ß, TNF-α and PGE_2_.

**Conclusion:**

The slices represent an instrument for investigating pharmacological interactions with udder tissue, which can be useful for studies on pharmacological questions and the understanding of complex pathophysiological processes of infection and inflammation.

**Supplementary Information:**

The online version contains supplementary material available at 10.1186/s12917-021-02817-w.

## Background

In view of the threatening global problem of antibiotic resistance, it is essential to intensify research on bacterial infectious diseases [1]. It is important to examine additional treatment options for infections caused by bacteria to ensure the efficacy of antibacterial agents in the future [2]. These options can offer new classes of antimicrobials or other agents. Investments in the development of new antibiotics have become more or less commercially attractive for pharmaceutical industries due to the increasing regulation and restriction of the use of antibiotics [3].

The principle of the 3 Rs and the promotion of the development and validation of alternatives to animal testing are enshrined in the EU directive 2010/63/EU. Various scientific approaches are already being used to investigate research questions in physiology or pathophysiology ranging from cell cultures on the one hand to in-vivo-experiments on the other. Precision-cut tissue models may serve as a bridge between cell culture and in-vivo-experiments. Various working groups demonstrated that precision-cut tissue slices can also successfully be used for experimental studies [4–6]. Among them, the model was applied to the lung or liver of various species, which is the reason why it has now been attempted to transfer the model to the bovine udder. In the cell association, studies on physiological and pathophysiological issues of the udder have so far been mainly carried out on explant cultures, where the tissue to be cultured was mostly obtained with scissors or a scalpel [7–9]. The size of the individual explants varied [9, 10]. In one study, udder tissue pieces of 2 mm^3^ were generated and stimulated with LPS, LTA and *S. aureus* [9]. The stimulation with LPS and *S. aureus* induced a significant increase in cytokine reaction. Another working group had the ability to show a possible stimulation on teat explants. They were able to show pathogen- and location-specific cytokine and chemokine reactions on these explants [8].

The topic of this study was the investigation of the suitability of precision-cut bovine udder slices (PCBUS) as an in-vitro-model for investigations of the early phase of bacterially caused inflammation. This should provide an in-vitro-model that allows the investigation of physiological as well as pathophysiological processes in the udder. It can be used for the research of different pharmacological agents and contribute to the development of alternative, efficient therapeutic approaches to minimize the use of antibiotics. In addition, the model should reduce the number of necessary in-vivo-studies.

## Results

### Viability

**Fig. 1:**
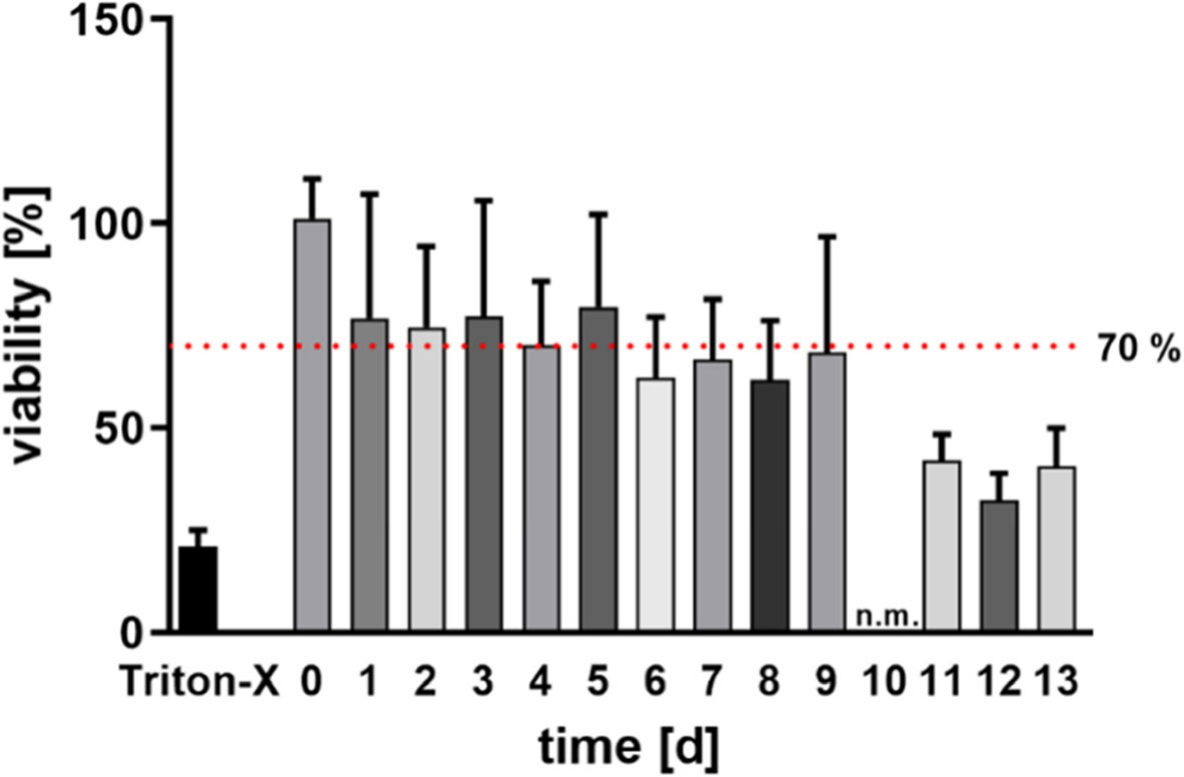
Viability of PCBUS over a period of 13 days; Triton X-100 represents the result of the negative control; the red dotted line symbolised a viability limit of 70%; n.m.: not measured; data are given as mean + standard deviation of 7 PCBUS from 4 udders each

To verify whether the cells of the udder tissue survived the process of generating PCBUS, an MTT assay was performed immediately after generating as well as every 24 h for as long as possible. Figure 1 shows the viability of PCBUS in the first viability study over a period of 0 to 13 days of incubation. As negative control Triton X-100 treated PCBUS showed a significant diminished viability until day 9. A limit of 70% of viability compared to time 0 (6 h after slaughter) was used as described in ISO 10993-5:2009 [11]. Derived from this, a period of 5 days was set for the main experiments. Only PCBUS with a viability of more than 70% compared with time 0 were used for stimulation experiments.

**Fig. 2:**
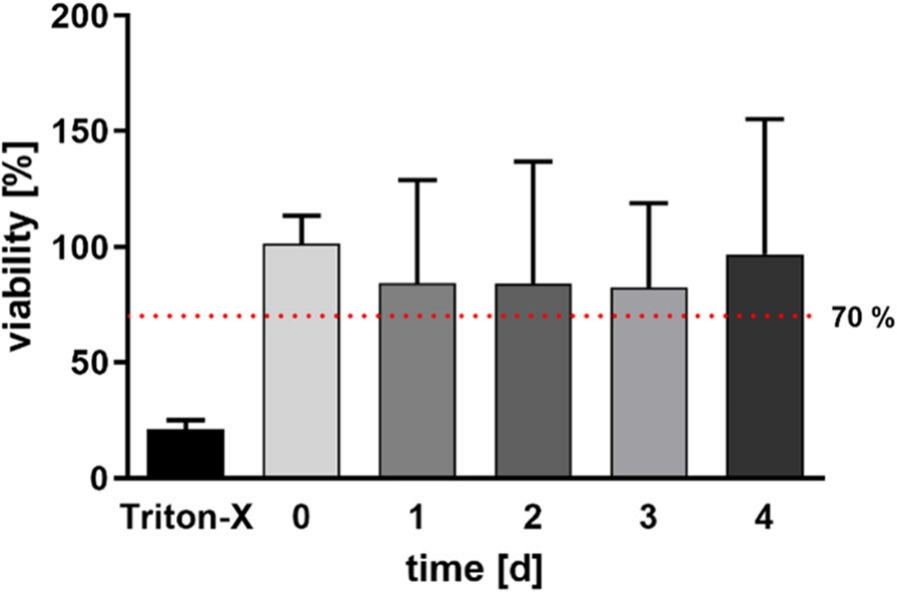
Viability of PCBUS, which were used in the stimulation experiment; Triton X-100 represents the result of the negative control; the red dotted line symbolized a viability limit of 70%; data are given as mean + standard deviation of 5 PCBUS from 6 udders each

Figure 2 shows the viability of the PCBUS which were used for stimulation tests. It should be noted that all PCBUS involved in the experiment had a viability of more than 70% compared with time 0 up to the end of the experiment.

### Morphology

**Fig. 3:**
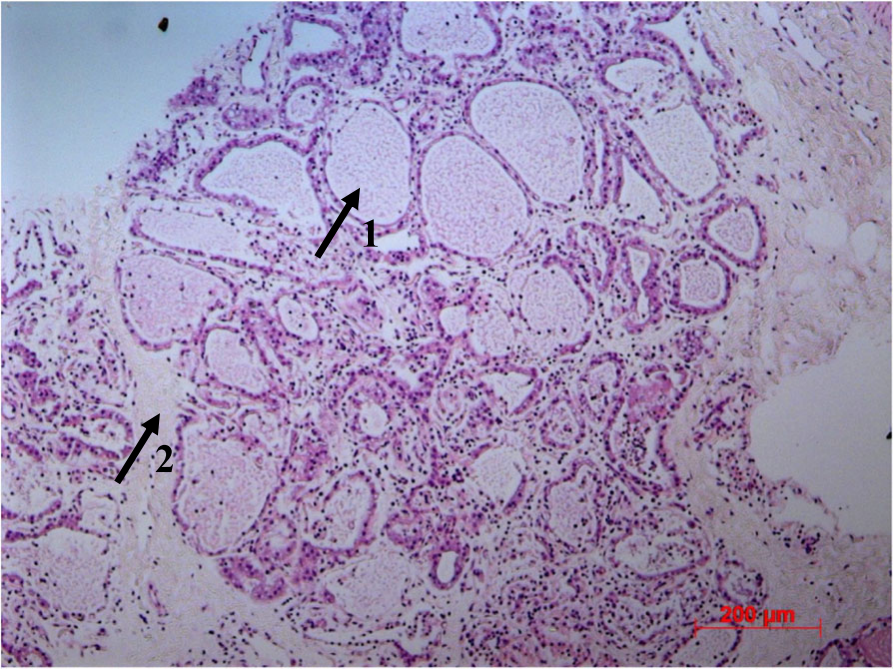
HE-staining of PCBUS shows physiological morphology of bovine udder; arrow 1 points to an alveolus; arrow 2 points to the interstitium; magnification 20 x

**Fig. 4:**
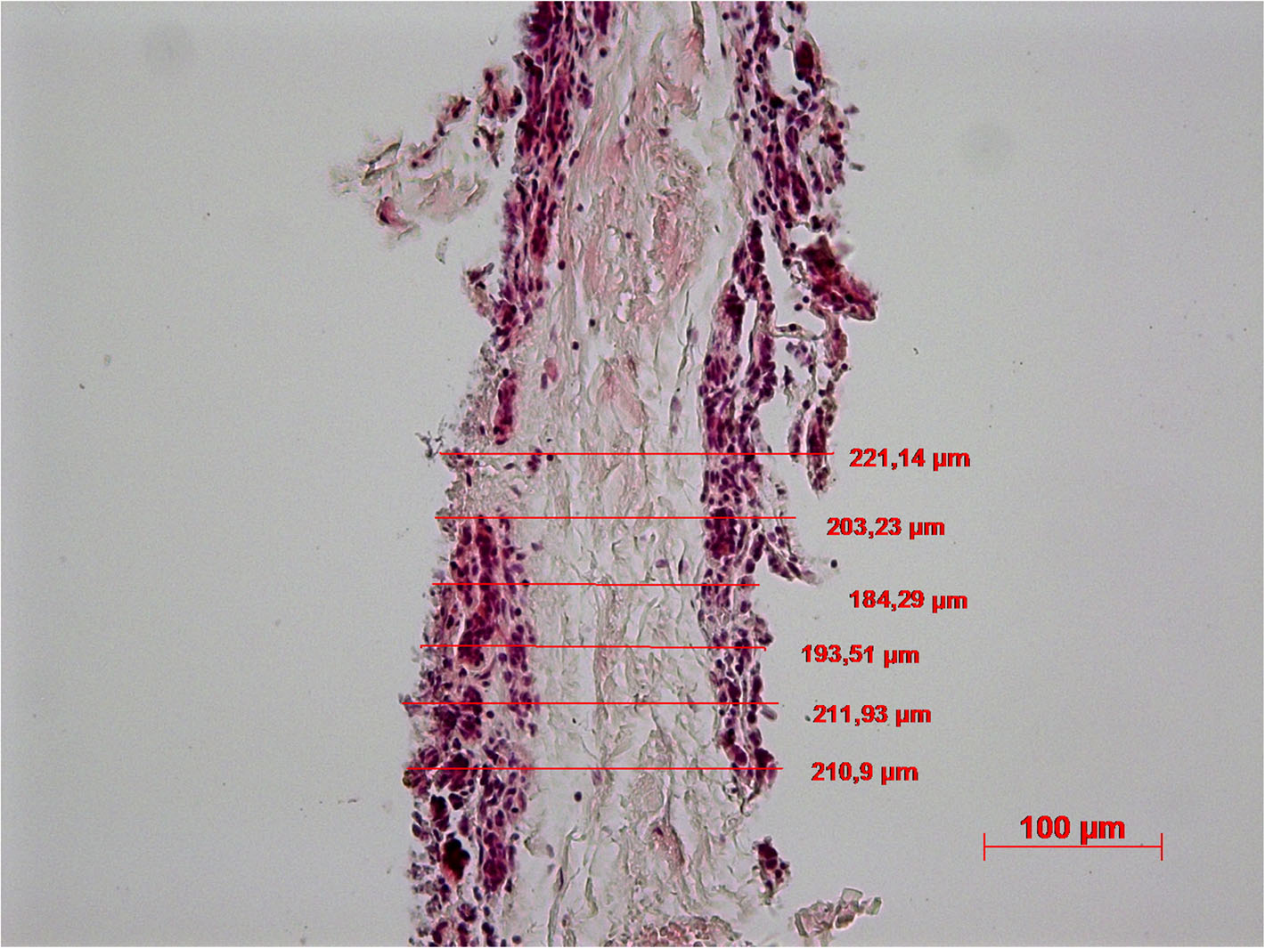
Histological section of a PCBUS (HE-staining); thickness measurement in μm; magnification 20 x

After PCBUS has been obtained, the morphology should be examined to ensure that the structure of the cells is not lost in this newly applied method. Figure 3 shows the typical histology of bovine gland tissue (magnification 20 x). Several alveoli filled with protein residues can be identified, which are separated from the lumen by epithelial cells. Intralobular milk ducts run in the interstitial connective tissue. The thickness of PCBUS deviated by ±15% from the target value of 250 μm as shown in Fig. 4 (magnification 20 x).

### Cytokine secretion

**Fig. 5:**
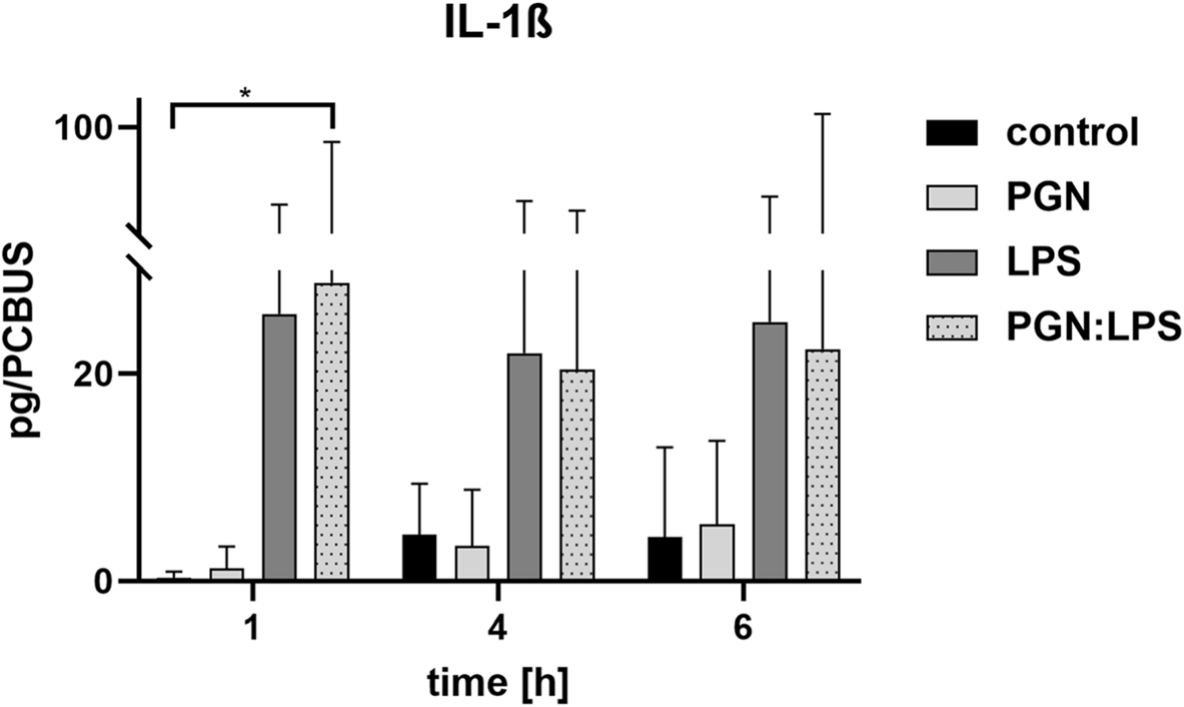
IL-1ß cytokine release profile after stimulation of PCBUS with PGN, LPS and the combination of both substances; performed with 3 PCBUS from 6 udders each; data are given as mean + standard deviation of 3 PCBUS from 6 udders each

To assess the possibility of stimulating cells from these PCBUS, we investigated their ability to respond to pro-inflammatory stimuli to which the mammary gland epithelium is exposed during bovine mastitis. We investigated cytokine levels in the supernatant of the experimental approaches for IL-1ß, PGE_2_ and TNF-α. Figure 5 shows the IL-1ß-release profile of PCBUS after stimulation with LPS (1 μg/mL), PGN (1 μg/mL) or a combination of both substances (1 μg/mL each) over time. A significant increase after 1 h is seen in PCBUS incubated with the combination of the two stimulants, while no significant increase in cytokine concentration can be seen after stimulation of PCBUS with the other substances.

**Fig. 6:**
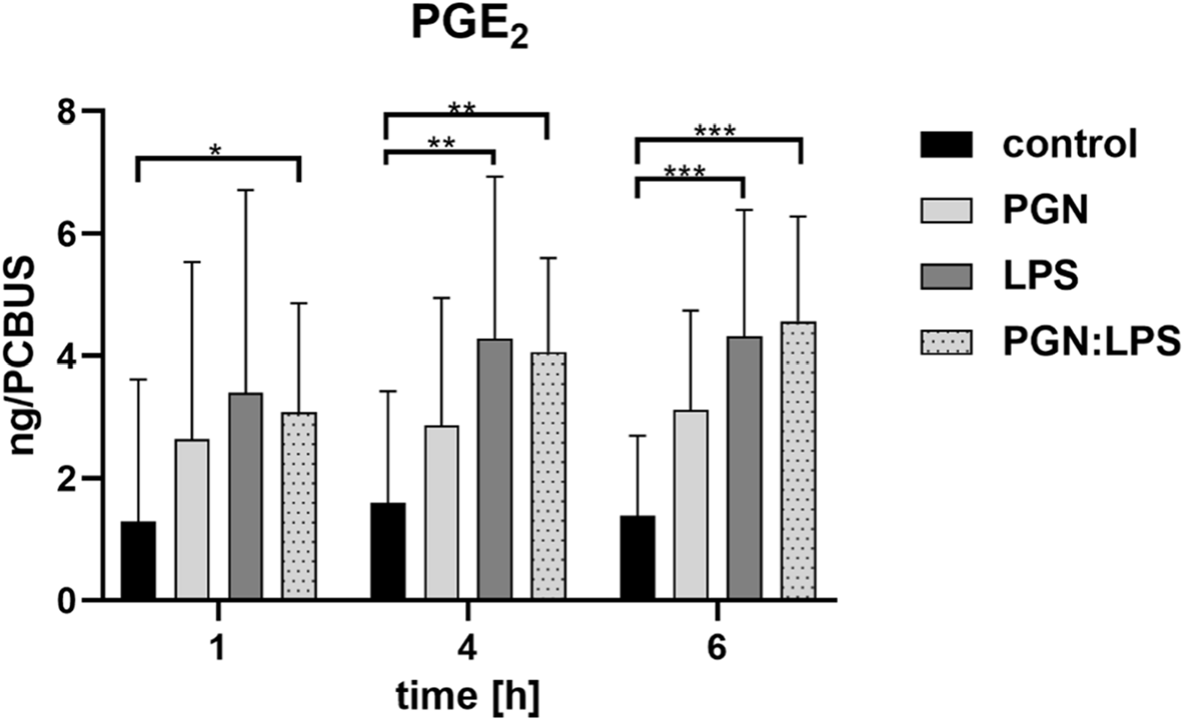
PGE_2_ release profile after stimulation of PCBUS with PGN, LPS and the combination of both substances; data are given as mean + standard deviation of 5 PCBUS from 6 udders each

A time-related increase in PGE_2_-concentrations is obvious in all stimulation approaches. Significant differences in PGE_2_-release was observed in PCBUS incubated with LPS and the combination (LPS:PGN) shown in Fig. 6.

**Fig. 7:**
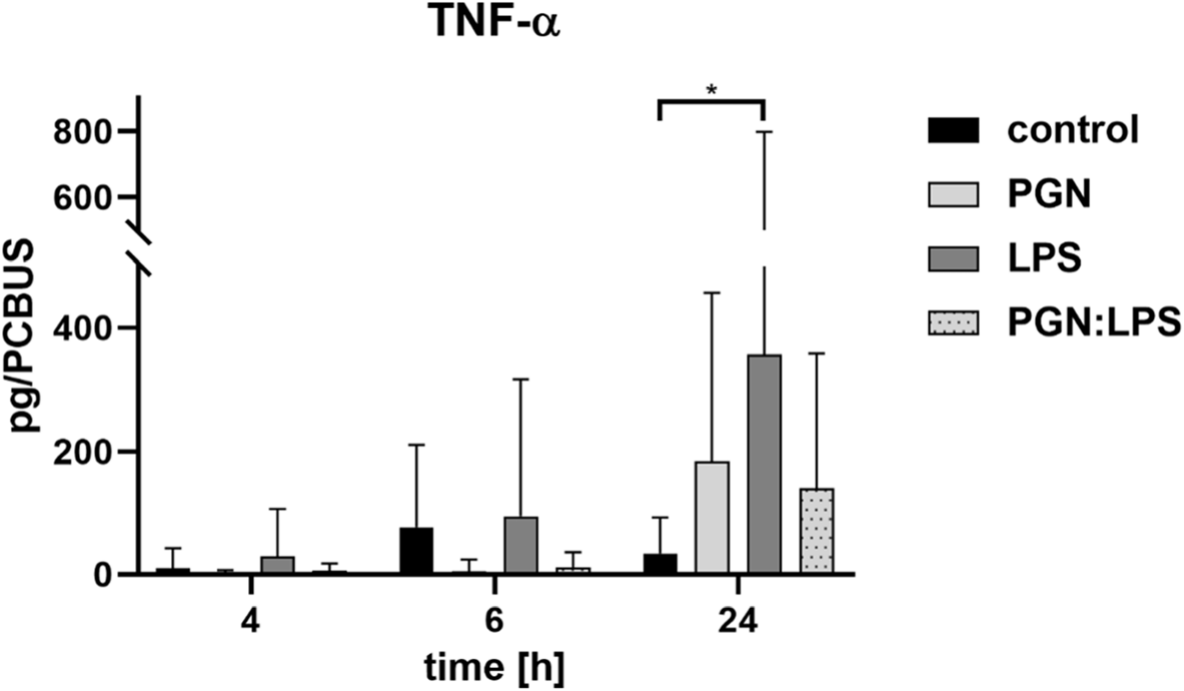
TNF-α cytokine release profile after stimulation of PCBUS with PGN, LPS and the combination of both substances; data are given as mean + standard deviation of 5 PCBUS from 6 udders each

A significant increase in TNF-α-release was detected 24 h after LPS-stimulation (Fig. 7).

## Discussion

### Aim of the study

Various scientific approaches were already been used to investigate the physiology and pathophysiology of inflammatory processes caused by infections as well as pharmacological interventions, ranging from cell culture experiments to in-vivo-studies [12–14]. A benefit of in-vitro-studies is the possibility to use larger numbers of replicates. This is rarely possible in ex-vivo- or in-vivo-experiments due to higher costs, lack of tissue availability and ethical issues [15]. It should be noted that there are also significant individual differences between genetically similar animals, requiring research on a large number of animals and consequently long and expensive experimental set-ups, having a greater impact on animal welfare [16]. Therefore, precision-cut tissue slices may be helpful to perform experimental studies before animal studies are performed. It was the aim of the present study to establish PCBUS as a possible replacement and complement to in-vivo-infection experiments.

### Tissue sources and preparation

In the described study, bovine udders of slaughtered dairy cows were used. In the past, it was demonstrated in several studies that slaughter material can be used to investigate different issues with reproducible results [17–19]. Although the udders were already adspectively and palpatorically examined on site and only the udders found to be healthy were selected for transport to the laboratory, in the laboratory additionally, the so-called California Mastitis Test (CMT) was performed with the milk of the supposedly healthy udder. Only udders whose cell count in the milk at the CMT was below 100.000 were included in the experiments. However, these examinations did not reveal totally whether there was a subclinical inflammation in the present organ. It should be helpful to get information about the stage of lactation of the slaughtered cows. Unfortunately, this was not possible in the present study. Because no preliminary report was available for the dissected udders, this is a limitation of the model.

When cutting the udder tissue, it became apparent that the udder quarters have a variable tissue structure and consistency. This interesting discovery mostly correlated with the size of the udder. In consequence, it was always important to adapt the method of obtaining PCBUS; the different lactation stages and the differences for milk in the udder required an adjustment of the number of washing steps to remove the remaining milk from the tissue. Based on the results of this feasibility study, it is therefore recommended that udder health (adspection, palpation, CMT result) and udder size (cow age, lactation stage) must to be taken into account when selecting the sample material. In future studies it is now possible, for example, to investigate differences between the individual lactation or age stages.

The measurement of the tissue slice thickness (Fig. 4) confirmed a good reproducibility with regard to the method of sample preparation. This shows the success of the cutting technique using the dermatome, which is important because tissue thickness can influence its cultivability and nutrient supply. Previous studies showed that precision-cut lung slices (PCLS) are better supplied with oxygen and nutrients at a section thickness of 250 μm [20] than the precision-cut lung slices generated with a thickness of 500–1000 μm [21].

An advantage of the described method is the generation of large number of PCBUS.

If the cutting and punching technique is correctly applied, more than 200 precision-cut udder slices can be obtained from one udder quarter. This allows simultaneous experimental approaches to the material and thereby originate from only one animal. In comparison to other studies, the technique used here has the advantage that the thickness of the samples [9] and thus the diffusion distance for nutrients and metabolites is smaller, resulting in a better exchange between tissue and environment, as already described for PCLS [20].

### Morphology

The histological examination of HE stained PCBUS confirms the typical morphology of bovine udder tissue (Fig. 3). This is consistent with results of previous studies [9], also identifying the morphological structure of bovine udder tissue from their udder explants. Our method not only allows the natural cell structure to be preserved, sections can also be studied in histological examinations, for example to check cell integrity after external stimuli. In addition, maintaining a certain slice thickness shows the high reproducibility of the model and ensures that all PCBUS are equally supplied with culture media.

### Viability

The viability of the PCBUS were determined by the MTT assay (Figs. 1 and 2). This test has already been used in numerous studies and is one of the standard tests for determining tissue or cell viability [22–24]. The ISO 10993-5:2009 standard [11] describes, that MTT assay results below 70% compared to 0 indicate reduced viability. Consequently, this limit was used in this work to exclude unsuited tissue slices. No PCBUS with viability of less than 70% were used in the experiments. However, it should be considered from which region of the udder the samples are generated, as the tissue composition is variable (ratio of gland tissue and connective tissue). Therefore, a sufficient number of PCBUS is necessary to minimize deviations.

The metabolic conversion of the dye during the MTT assay is done by the mitochondrial dehydrogenases of the gland cells, in the connective tissue of PCBUS the dye cannot be produced by corresponding cells. So, if the ratio of glandular to connective tissue in the individual PCBUS is different, the result of this test could fluctuate. However, the PCBUS obtained from one udder showed for the most part a similar tissue structure, so that a similar ratio of glandular to connective tissue is assumed.

Nevertheless, the PCBUS could be reliably cultivated and maintained in a viable condition for more than 96 h, regardless of the tissue composition.

### Early inflammatory reaction

LPS and PGN were used as stimulants for the experiment, as these are cell wall components of Gram-negative and Gram-positive bacteria, respectively, which have an immunostimulating effect, thereby recruiting immune cells and inducing the release of cytokines [14, 25, 26]. The feasibility of the experimental setup was confirmed by the fact that stimulation with LPS or PGN was possible. The results of a stimulation by LPS and PGN (1 μg/mL each) showed an enhanced secretion of IL-1ß, TNF-α and PGE_2_ after stimulation. After stimulation of PCBUS with LPS, a significant release of IL-1ß as well as PGE_2_ can be seen after only 1 h and is still visible after 4 and 6 h. In contrast to the control, the release of PGE_2_ is even significant after 4 and 6 h. A combination of the two stimulants (LPS:PGN) even led to a significant release of PGE_2_ after only 1 h, until the end of the study period. In comparison, the release of IL-1ß was significant only after 1 h. We also expected a significant increase in IL-1ß release at the other measurement points in contrast to the control. However, this could not be observed, which we explained after the first investigations by the fact that also the cells of the control PCBUS start to become apoptotic and increase the IL-1ß concentration. This requires further attention in future studies and needs to be observed more closely. In contrast, a significant release of TNF-α could only be shown after stimulation with LPS 24 h. The stimulation of PCBUS with PGN did not show a significant increase in any of the mediators, investigated at any time period considered. It must of course be considered that cell types are confronted with the stimulants that would not encounter the stimuli in vivo. In order to study cells that are only present in the lumen or milk, a different model should be chosen, e.g. the isolated perfused udder [18]. Therefore, the PCBUS model focuses on the large time slot, which allows longer-term examinations than the isolated perfused udder, where only studies of 8 h maximum are possible.

It can be concluded, that the sections are useful to study the effect of stimulating agents. Further investigations can provide information on how PCBUS react to a bacterial infection.

## Conclusion

Advantages of this method are the very high reproducibility of the technique of obtaining tissue sections of uniform size, kept viable over a large time slot for stimulation and thus in the future also for infection experiments. In addition, it is possible to preserve the physiological cell structure and thus histological examinations of the morphology in physiological and pathophysiological stages. The differentiated immunological response of PCBUS to different stimulants gives reasons to hope that in the future the processes of bovine mastitis can be simulated in vitro and thus new therapeutic options can be investigated. Thus, it is interesting for future studies to investigate a larger gene spectrum of innate immunity, e.g. with RT-qPCR or RNA sequence analysis. Furthermore, there are some variations in our collected data that can be explained by the procurement of the sample material. Since the udders are obtained from the slaughterhouse, we do not have any preliminary reports on the animals. Therefore, we do not know whether the animal suffered from subclinical mastitis at the time of slaughter and what the immune status of the animal was. In sum, the described methodology makes it possible to generate precision-cut bovine udder slices (PCBUS), may be used for experimental pharmacological studies in the future.

## Methods

### Tissue sources and preparation

**Fig. 8:**
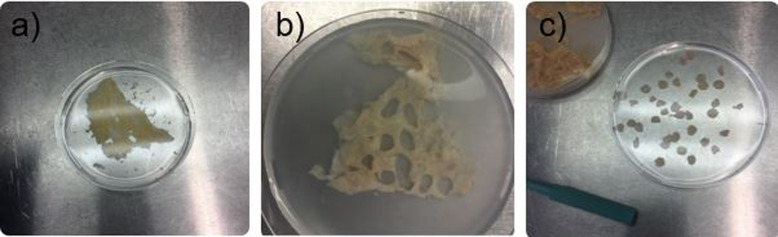
**a** glandular tissue of the udder after using a dermatome with a layer thickness of 250 μm in a petri dish with sterile PBS; **b** glandular tissue after punching; **c** obtained PCBUS with a diameter of 6 mm

Before the preparation of precision-cut bovine udder slices (PCBUS), udders of slaughtered cows (German Holstein Friesian) were immediately examined for udder health by adspection and palpation. The dissected udder was infused by heparinized tyrode solution right away to prevent clot formation in the vessels [18] and instantly transported to the laboratory. In the laboratory, we performed the so-called California Mastitis Test (CMT) with the milk of the supposedly healthy udder. The CMT is used to determine the cell count in the milk, which is an additional indicator of udder health. A piece of approximately 10 × 10 × 5 cm was cut out with a sterile scalpel blade. A dermatome (Zimmer U.K. Ltd., Swindon, United Kingdom) was used to get tissue slices with a thickness of 250 μm (see supplementary video 1, Additional file 1). The obtained tissue slices were rapidly washed in sterile phosphate-buffered saline (pH = 7.4; PBS) until a clear solution without milk contamination was achieved. Up to 200 PCBUS biopsy punches (6 mm diameter) were than taken from the slices (Fig. 8), transferred to 24-well plates and washed with RPMI-1640 medium (Biochrom GmbH, Berlin, Germany), supplemented with 20% fetal calf serum (FCS; Biochrom GmbH), 10% penicillin streptomycin (10,000 international units (I.U.)/mL/ 10,000 μg/mL, Biochrom GmbH), 10% amphotericin B (PAA, Laboratories GmbH, Pasching, Austria) and 15 μg/mL gentamicin (PAA Laboratories GmbH) on an orbital shaker (130 rpm) in accordance to Magro et al. [9]. Two additional washing steps were performed with RPMI-1640-medium, supplemented with 10% FCS, 1% penicillin streptomycin, 1% amphotericin B and 15 μg/mL gentamicin (maintenance medium). The slices were put into a new 24-well plate (Greiner Bio-One, Frickenhausen, Germany) and incubated in 1 mL of maintenance medium in a humidified atmosphere containing 5% CO_2_ at 37 °C. Incubation was performed for 13 days totally and the medium was changed every 48 h.

### Viability of PCBUS

In first experiments with four udders, viability of the PCBUS was tested from time point 0 h (6 h after slaughtering) until day 13, whereby the medium was changed every 48 h. For this purpose, the MTT assay was performed daily with 7 PCBUS of the respective udder. The MTT assay as described by Lambermont et al. [25] was used with some modifications. The PCBUS were transferred into a 24-well plate with 900 μL RPMI-1640-medium and 100 μL 3-(4,5-dimethylthiazol-2-yl)-2,5-diphenyl tetrazolium bromide solution (MTT, 7 mg/mL; Sigma-Aldrich, Steinheim, Germany). After 15 min of incubation, the supernatant was discarded and 200 μL of an intermixture (5% formic acid + 95% propanol; Sigma-Aldrich) was added. After a further 40 min, 100 μL supernatant were transferred to a 96-well plate (Greiner Bio-One) for extinction measurement at 570 nm (MRX-reader, Dynatech, Denkendorf, Germany). As negative control, PCBUS were digested with Triton X-100 (Sigma-Aldrich; 1 mL, 30 min, 37 °C, 5% CO_2_ atmosphere) 30 min before starting the MTT assay. All steps were carried out at room temperature in the dark. The main trials (stimulation trials) were performed over a period of 5 days. Therefore, 5 PCBUS of each of the 6 udders used in the trials were checked daily by the MTT assay for their viability, until the end of the experiments.

### Stimulation of PCBUS with LPS and PGN

One day before stimulation, PCBUS were incubated in RPMI-1640-medium, supplemented with 10% FCS, but without any antibiotics, in a humidified atmosphere containing 5% CO_2_ and 37 °C. To simulate a pathophysiological reaction, initiated by bacterial toxins during early phase of infection, 1 μg/mL lipopolysaccharide (LPS; Sigma-Aldrich) of *E. coli* O55:B5, 1 μg/mL peptidoglycan (PGN from *S. aureus*, Sigma-Aldrich) and a combination of LPS and PGN was added to the incubation medium. As a control, unstimulated PCBUS were used. These PCBUS were incubated with RPMI-1640-medium, supplemented with 10% FCS. One hour and two hours post stimulation, 30 μL supernatant and after 4 h, 6 h and 24 h, 130 μL supernatant were sampled. After removal of the samples, the wells were refilled with fresh medium (RPMI-1640-medium + 10% FCS; without any stimulants). The experiments were performed in triplicates from 6 different udders.

Tissue slices sampled the same time points and were used for histological examination from other wells.

**Fig. 9:**
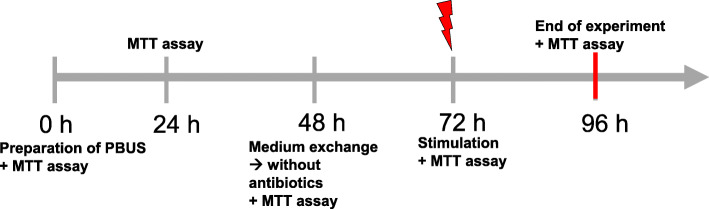
Overview of the chronological course of the experiment

**Fig. 10:**
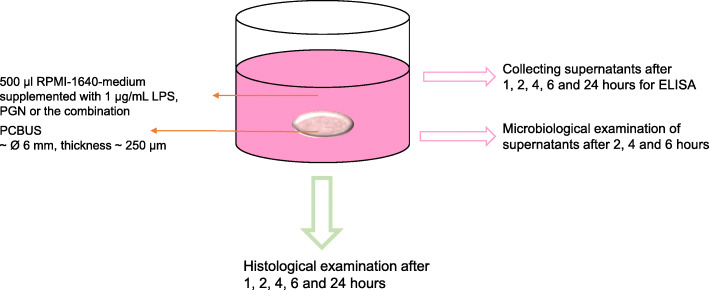
Overview of sample collection at different time points

A graphical representation of the experimental setup and sample collection is shown in Figs. 9 and 10.

### Measurement of IL-1ß, TNF-α and PGE_2_

Concentrations of IL-1ß, TNF-α and PGE_2_ in supernatants were measured with ELISA kits, according to the manufacture’s specification (bovine Interleukin-1ß Reagent Kit Invitrogen, Thermo Fisher Scientific; Prostaglandin E2 Express ELISA Kit Cayman Chemical Company, Ann Arbor, USA; Bovine TNF-alpha DuoSet® ELISA DuoSet® ELISA Development Systems, R&D Systems, Minneapolis, USA). Dilutions of the samples were determined in preliminary tests. For the determination of the IL-1ß- as well as PGE_2_-concentrations, the samples were diluted 1:10 according to the manufacturer’s instructions. For the determination of the TNF-α content, the samples were applied undiluted.

### Histological examination

For histological examination, PCBUS were fixed in 10% buffered formalin for 48 h and embedded in paraffin wax, using standard techniques. Three micrometres slices were stained with hematoxylin eosin staining (HE staining). All sections were examined by light microscopy. The thickness of PCBUS were measured by using computer-aided software (AxioVision Software).

### Statistical analysis

The results are presented as mean ± standard deviation (SD). The statistical evaluation was performed with GraphPad Prism (Version 8.0.1, GraphPad Software, Inc.). In the experiments, the results of the control groups at the respective measurement time were compared with those of the stimulated group, with regard to significant differences, using the Kruskal-Wallis test. The significance of error probability of 5% was considered significant, with significances of ≤0.01 and ≤ 0.001 being particularly marked in the figures.

## Supplementary Information

**Additional file 1.**

## Data Availability

The datasets used and/or analysed during the current study are available from the corresponding author on reasonable request.

## Abbreviations

CMT: California mastitis test
*E. coli*: *Escherichia coli*
FCS: Fetal calf serum
HE: Hematoxylin eosin
IL-1ß: Interleukin-1ß
LPS: Lipopolysaccharide
LTA: Lipoteichoic Acid
MTT: 3-(4,5-dimethylthiazol-2-yl)-2,5-diphenyltetrazolium bromide
PCBUS: Precision-cut bovine udder slices
PCLS: Precision-cut lung slices
PGE_2_: Prostaglandin E_2_
PBS: Phosphate-buffered saline
*S. aureus*: *Staphylococcus aureus*
TNF-α: Tumor necrosis factor-α
